# Barriers and Facilitators for Bringing Model‐Informed Precision Dosing to the Patient's Bedside: A Systematic Review

**DOI:** 10.1002/cpt.3510

**Published:** 2024-12-10

**Authors:** Anna Caroline Dibbets, Charlotte Koldeweij, Esra P. Osinga, Hubertina C. J. Scheepers, Saskia N. de Wildt

**Affiliations:** ^1^ Division of Pharmacology and Toxicology, Department of Pharmacy Radboud University Medical Center Nijmegen The Netherlands; ^2^ Department of Obstetrics and Gynaecology Maastricht University Medical Center Maastricht The Netherlands; ^3^ GROW, Institute for Oncology and Reproduction Maastricht The Netherlands; ^4^ Department of Pediatric and Neonatal Intensive Care Erasmus MC‐Sophia Children's Hospital Rotterdam The Netherlands; ^5^ Department of Intensive Care Radboud University Medical Center Nijmegen The Netherlands

## Abstract

Model‐informed precision dosing (MIPD) utilizes mathematical models to predict optimal medication doses for a specific patient or patient population. However, the factors influencing the implementation of MIPD have not been fully elucidated, hindering its widespread use in clinical practice. A systematic review was conducted in PubMed from inception to December 2022, aiming to identify barriers and facilitators for the implementation of MIPD into patient care. Articles with a focus on implementation of MIPD were eligible for this review. After screening titles and abstracts, full articles investigating the clinical implementation of MIPD were included for data extraction. Of 790 records identified, 15 publications were included. A total of 72 barriers and facilitators across seven categories were extracted through a hybrid thematic analysis. Barriers comprised limited data for model validation, unclear regulatory pathways for model endorsement and additional drug level measurements required for certain types of MIPD. Facilitators encompassed the development of user‐friendly MIPD tools continuously updated based on user feedback and data. Collaborative efforts among diverse stakeholders for model validation and implementation, along with education of end‐users, may promote the utilization of MIPD in patient care. Despite ongoing challenges, this systematic review revealed various strategies to facilitate the clinical implementation of MIPD.

**Study Highlights**

**WHAT IS THE CURRENT KNOWLEDGE ON THE TOPIC?**

Model‐informed precision dosing (MIPD) utilizes mathematical models to predict the optimal medication dose tailored to an individual patient or a patient population. The goal of MIPD is to enhance drug treatment by determining the most adequate dose to achieve therapeutic benefits, while preventing toxicity. However, little research has been conducted on the clinical implementation of MIPD, which remains limited in practice.

**WHAT QUESTION DID THIS STUDY ADDRESS?**

This systematic review aimed to identify barriers and facilitators for the implementation of MIPD into patient care.

**WHAT DOES THIS STUDY ADD TO OUR KNOWLEDGE?**

This study draws an overview of barriers and facilitators pertaining to the implementation of MIPD in clinical care. It underscores key challenges that need to be addressed, including unclear regulatory pathways for model endorsement, limited data availability for model validation, technical and logistical hurdles, and financial barriers linked to implementing MIPD in clinical practice. Additionally, it highlights opportunities for promoting MIPD implementation, emphasizing the importance of multi‐stakeholder collaboration and awareness‐raising regarding the benefits of MIPD.

**HOW MIGHT THIS CHANGE CLINICAL PHARMACOLOGY OR TRANSLATIONAL SCIENCE?**

We have highlighted important barriers for the clinical application of MIPD and potential opportunities to address them. This may promote a broader use of MIPD in clinical care, with potential improvement in drug therapies for special populations.

Model‐informed precision dosing (MIPD) utilizes mathematical models to predict optimal medication doses considering specific patient characteristics such as age, weight, and comorbidities.

MIPD has emerged as an alternative approach for dosing alongside empirical methods that have historically been used to determine medication doses on drug labels.^1^ In contrast with a one‐size‐fits all approach to dosing, the use of MIPD is gaining traction given its potential to achieve enhanced therapeutic outcomes and reduced toxicity for individual patients.^2^ MIPD may be particularly advantageous in cases where physiological variations may necessitate dose adjustments and where data to support adequate dosing are limited.^3^ This is particularly true for so‐called “special populations” such as children, pregnant women or adults with renal or hepatic impairment, whose physiological characteristics may differ from healthy adults. However, MIPD may only be beneficial when a clear dose–response relationship and exposure–safety relationship is present.^4^

MIPD encompasses a variety of approaches ranging from dosing recommendations for patient groups (“population‐level MIPD”) to doses tailored to individual patients. Population‐level MIPD involves providing dose recommendations for a group of patients who share certain characteristics. These fixed, population‐level doses are generally established and/or endorsed prior to clinical use. For instance, a model‐informed dosing strategy may be determined for pregnant women, considering their altered pharmacokinetic and pharmacodynamic profiles compared to nonpregnant adults, requiring dose adjustments for certain medications.^5^, ^6^ These group‐level recommendations can further vary based on factors like gestational age, but do not require reliance on extensive calculations, or modeling and simulations at the point of care.

Various models can be used to establish dose recommendations at the population level. These include population pharmacokinetic (popPK) and physiologically‐based pharmacokinetic (PBPK) models that integrate population and/or physiological data with drug characteristics to predict drug exposures for a given dose, and pharmacokinetic‐pharmacodynamic (PK‐PD) models linking drug concentration and effect.^7^, ^8^, ^9^ These models have for example supported dose recommendations for several antibiotics in critically ill children, and for the antiretroviral darunavir during pregnancy.^10^, ^11^ Population‐level models require validation, which may be challenging given limited data in certain patient populations.^2^

Population‐level MIPD is already being used to inform dose recommendations in drug labeling. The use of models can help optimize clinical trial designs by guiding the selection of potentially successful dosing regimens.^12^ In the later phases of drug development, modeling helps to characterize variability in drug concentrations and responses.^13^ Population‐level MIPD can also inform off‐label dose recommendations in clinical practice.^14^

In contrast, personalized MIPD approaches integrate individual patient data, such as body weight, pharmacogenetic information or other relevant patient covariates, to determine the optimal (starting) dose for an individual patient at the point of care. By integrating information on the pharmacokinetics and pharmacodynamics of a drug with patient‐specific characteristics, such as age and renal function, personalized MIPD can predict the optimal medication dose for an individual patient.^2^, ^15^, ^16^, ^17^ Data from a specific patient can be derived from various models, such as popPK, PBPK, and PK/PD. For instance, a popPK model incorporating age, body surface area and cytochrome P450 genotypes was used to determine the initial tacrolimus dose for adult renal transplant recipients.^18^

Moreover, personalized MIPD can be combined with therapeutic drug monitoring (TDM) to support dose adjustments a posteriori, a practice that may be referred to as “individualized MIPD.”^4^, ^17^, ^19^ Subsequent adjustments are made based on drug concentrations or biomarkers from the patient, using Bayesian methods to estimate optimal doses, from individual pharmacokinetic and pharmacodynamic parameters.^20^ Kantasiripitak et al. investigated the use of individualized MIPD for infliximab dosing in patients with inflammatory bowel disease.^21^

The use of personalized MIPD necessitates specific patient data, such as pharmacogenetic information, to estimate individual doses, which may be time‐consuming and/or costly. Individualized MIPD introduces additional complexity and infrastructure requirements, as it requires taking drug concentration measurements as well as integrating these data into the model used for subsequent dosing.^20^, ^22^

Population‐level and personalized or individualized MIPD approaches may be seen as part of a continuum ranging from fully standardized, fixed doses to highly individualized dosing integrating multiple patient characteristics obtained at the point of care. These approaches differ in their scientific and logistical requirements, resulting in varying degrees of specificity and complexity, as illustrated in **Figure** **1**.

**Figure 1:**
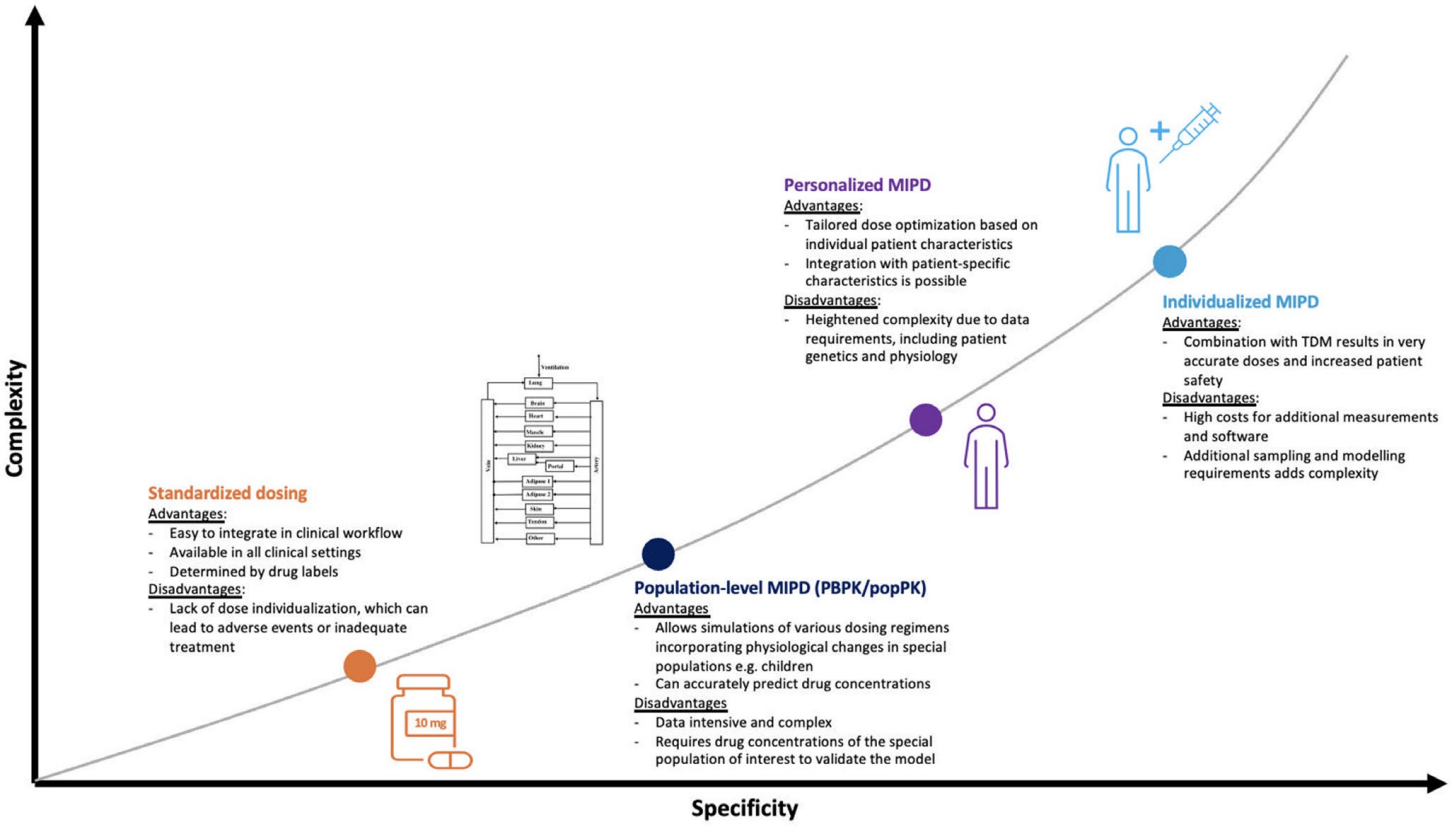
Specificity and complexity of various examples of model‐informed precision dosing approaches. CDS, clinical decision support; MIPD, model‐informed precision dosing; PBPK, physiologically‐based pharmacokinetic modeling; PK, pharmacokinetic; PopPK, population‐pharmacokinetics; TDM, therapeutic drug monitoring.

Although rapidly growing, the available literature on MIPD primarily focuses on model development or validation.^23^, ^24^ Meanwhile, the use of MIPD in clinical settings is often restricted to local initiatives in academic hospitals.^2^ Overall, research on the practical utilization of MIPD approaches remains limited.^2^, ^19^ This knowledge gap precludes patients from accessing the potential benefits of MIPD in enhancing treatment outcomes. Our systematic review aims to identify barriers and facilitators for the clinical implementation of MIPD, including population‐level, personalized and individualized MIPD approaches.

## METHODS

### Search strategy

We followed the 2020 Preferred Reporting Items for Systematic Reviews and Meta‐Analyses (PRISMA) guidelines to conduct this systematic review.^25^ A literature search was conducted in PubMed using a search string combining three groups of search terms (**Table** **S1**). The first group comprised MIPD and synonyms, such as “precision dosing” and “model‐based dosing,” together with “PBPK” or “popPK models.” The second element covered clinical care, including search terms such as “patient” and “healthcare.” The final element described implementation, using terms like “barriers,” “facilitators,” and “opportunities.” The search string was verified by a librarian from the Radboud University Medical Center. The search was carried out on December 12, 2022, with no restrictions in publication years. Additional studies were identified by consulting the references of the included studies (“snowballing”).

### Eligibility criteria

Eligible studies should report on the clinical implementation of MIPD. Additionally, studies investigating or economic considerations for MIPD implementation in clinical care were eligible for inclusion. Only published studies in English were considered for inclusion. There were no restrictions on study design or model type being used for MIPD. Studies were excluded if they mainly focused on model development, validation, performance or prediction, drug development or drug–drug interactions. Articles focusing on clinical decision support (CDS) tools or TDM without the use of pharmacokinetic (PK) models were also ineligible for this review.

### Study selection

**Figure 2:**
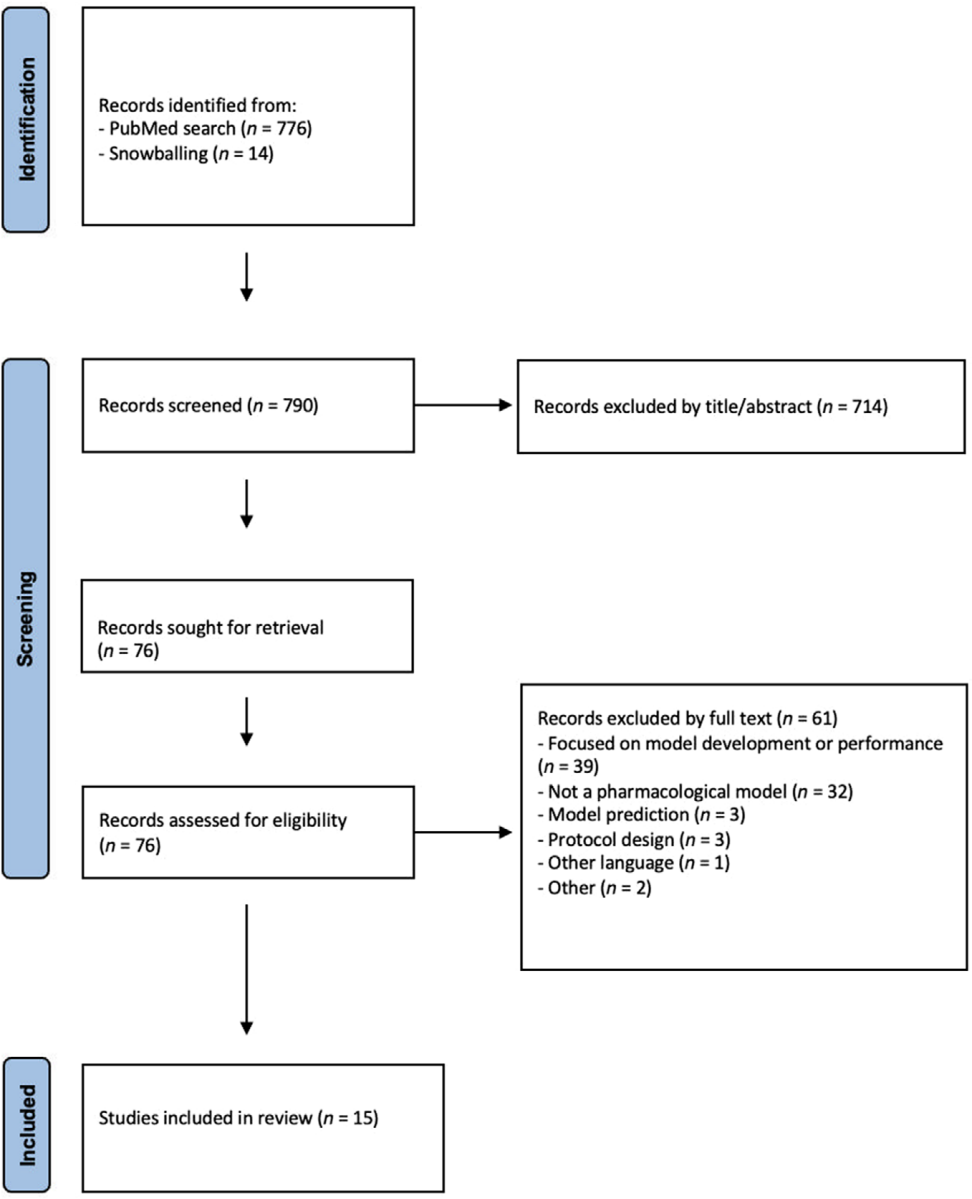
PRISMA diagram of study selection.^25^

Articles obtained from the database search and snowballing were combined. Initial screening of articles involved two independent reviewers (CD, PhD candidate in pharmacology and EO, master's student in medical biology). Article titles and abstracts were assessed for eligibility. Potentially eligible articles underwent a full‐text review, and those that did not meet eligibility criteria were excluded (**Figure** **2**). Disagreements between reviewers regarding eligibility were solved by consulting a third reviewer (CK, medical doctor, social scientist, and PhD candidate in pharmacology).

### Data extraction

Both reviewers independently extracted data from each article, including article type, MIPD approach, medication, setting, population, and implementation aspects. A barrier was defined as a factor hindering MIPD implementation or making it more difficult, while a facilitator was defined as a factor supporting or promoting implementation. A hybrid thematic analysis was conducted by two researchers (CD and EO), who independently identified all barriers and facilitators. Discrepancies were resolved through discussion. Barriers and facilitators were extracted for each MIPD approach. The barriers and facilitators were then categorized into domains, categories, and subcategories by CD and EO. These categories were derived from Sluisveld et al.'s^26^ framework, itself based on three frameworks from implementation science.^27^, ^28^, ^29^ Certain domains and categories within the framework were merged or adapted to better align with the data. In addition, the framework was expanded with subcategories identified through inductive analysis of the barriers and facilitators identified in the included articles. Any differences were resolved through discussion and consultation with CK as a third reviewer.

### Assessment of study quality

A quality appraisal of included studies was performed following the Joanna Briggs Institute (JBI) critical appraisal checklists. Given the inclusion of publications with various study designs, different critical JBI appraisal checklists were used.^30^ The checklists assessed the internal validity and risk of bias for each article type, in addition to clear reporting. Each included study was evaluated by two reviewers (CD and EO). Any uncertainties or disagreements on study quality scoring were resolved through consensus.

## RESULTS

### Study selection

The search strategy identified 790 unique articles. Of these 790 articles, 714 articles were excluded based on title and abstract, resulting in 76 full‐text articles being assessed for eligibility (**Figure** **2**). Fifteen articles were included for data extraction (**Table** **1**). Articles were most frequently excluded because they did not primarily center on model implementation or examined aspects outside the scope of this review, such as model development or performance evaluation.

**Table 1:** Characteristics of eligible studies

Study	Article type	Population or individualized MIPD	Medication	Setting	Population	Implementation aspects
Darwich et al. (2017)^2^	Narrative review	Individualized MIPD^a^ Personalized MIPD Population‐level MIPD	Not specified	General	General	Implementation in clinical care: Model validation Cost‐effectiveness Requirements for MIPD tools
Euteneuer et al. (2019)^31^	Narrative review	Individualized MIPD^a^ Population‐level MIPD	Examples: fluconazole, acetaminophen, morphine	General	Pediatrics (neonates)	Implementation in clinical care: Integration in EHR
Frymoyer et al. (2020)^32^	Original research	Individualized MIPD^a^ Personalized MIPD Population‐level MIPD	Vancomycin	Academic children's hospital	Pediatrics	Implementation in clinical care: TDM for dose validation Integration in EHR
Gonzalez et al. (2017)^33^	Narrative review	Individualized MIPD Personalized MIPD^a^ Population‐level MIPD	Not specified	General	General	Development and validation of MIPD tools and implementation in clinical care: TDM for dose validation Integration in EHR
Kantasiripitak et al. (2020)^22^	Original research	Individualized MIPD^a^ Personalized MIPD Population‐level MIPD	Not specified	General	General	Implementation in clinical care: Model validation Integration in EHR Cost‐effectiveness User interface software
Keizer et al. (2018)^34^	Perspective	Individualized MIPD^a^ Personalized MIPD Population‐level MIPD	Not specified	General	General	Implementation in clinical care: Model selection Model qualification
Kluwe et al. (2020)^20^	Perspective	Individualized MIPD^a^ Personalized MIPD Population‐level MIPD	Not specified	General	General	Implementation in clinical care: Multistakeholder collaboration User‐friendliness Implementation strategies
Long‐Boyle et al. (2015)^35^	Original research	Population‐level MIPD	Busulfan	Children's hospital	Pediatrics and young adults	Implementation in clinical care: User friendliness
Maier et al. (2022)^18^	Original research	Individualized MIPD	Paclitaxel‐induced neutropenia/ general	General	General	Implementation in clinical care: TDM for dose validation Continued learning approach
Maxfield et al. (2020)^36^	Perspective	Population‐level MIPD^a^ Personalized MIPD	Not specified	General	General	Implementation in clinical care: Integration in EHR Integration in CDS
Mizuno et al. (2022)^16^	Narrative review	Individualized MIPD^a^ Population‐level MIPD	Morphine, methotrexate, hydroxyurea, and sirolimus	General	General	Implementation in clinical care: Integration in EHR
Perry et al. (2020)^15^	Literature review	Population‐level MIPD	FDA‐approved drug products in therapeutic fields.	General	General	Implementation in clinical care for different therapeutic areas
Polasek et al. (2019)^37^	Meeting report (symposium)	Individualized MIPD Personalized MIPD Population‐level MIPD^a^	Not specified	General	General	Regulatory approval and implementation in and beyond clinical care
Polasek, Shakib et al. (2019)^38^	Perspective	Personalized MIPD Population‐level MIPD^a^	Not specified	General	General	Implementation in clinical care: Model validation Integration in CDS
Vinks et al. (2020)^39^	Narrative review	Individualized MIPD	Not specified	General	General	Implementation in clinical care: Integration in EHR

CDS, clinical decision support; EHR, electronic health records; MIPD, model‐informed precision dosing; TDM, therapeutic drug monitoring.

^a^ Primary focus of the article.

### Study characteristics

Included articles were published between 2015 and 2022. Included study designs comprised narrative reviews, expert opinions or perspectives, and original research, including three observational studies and a survey. Thirteen articles primarily focused on personalized or individualized MIPD; the remaining two articles mainly examined population‐level MIPD. Most studies (*n* = 12) examined the implementation of MIPD within a general patient population, while the remaining studies centered on pediatric patients. A majority of the studies (*n* = 9) investigated the application of MIPD in a general context rather than for a specific medication. Sampled studies explored various aspects of MIPD implementation in clinical care, including the integration of MIPD in Electronic Health Records (EHR), TDM, CDS, and considerations regarding cost‐effectiveness and user‐friendliness of user‐facing interfaces.

### Identified barriers and facilitators

Seventy‐two barriers and facilitators were identified, which were classified in three domains: innovation, users and stakeholders, and implementation. These domains were divided into seven categories, which were further subdivided into 20 subcategories (**Table** **2**).

**Table 2:** Domains, categories, and subcategories identified through thematic data analysis

Domain	Category	Subcategory
Innovation	Credibility & verifiability	Quality of evidence and model certainty Complexity
Users and stakeholders	Attitude	HCP mindset Collaboration
	Awareness‐raising and education	Comprehensibility Knowledge‐building
	Work routine	
Implementation	Relevance	Medication selection Clinical value
	Feasibility	Regulatory aspects Available resources Economic feasibility Sustainability
	Acceptability	Evidence generation Information for HCPs Patient information Safety safeguards Quality improvement
	Access & usability	Hosting application Integration in health technology

HCP, healthcare practitioner.

### Study findings

**Figure 3:**
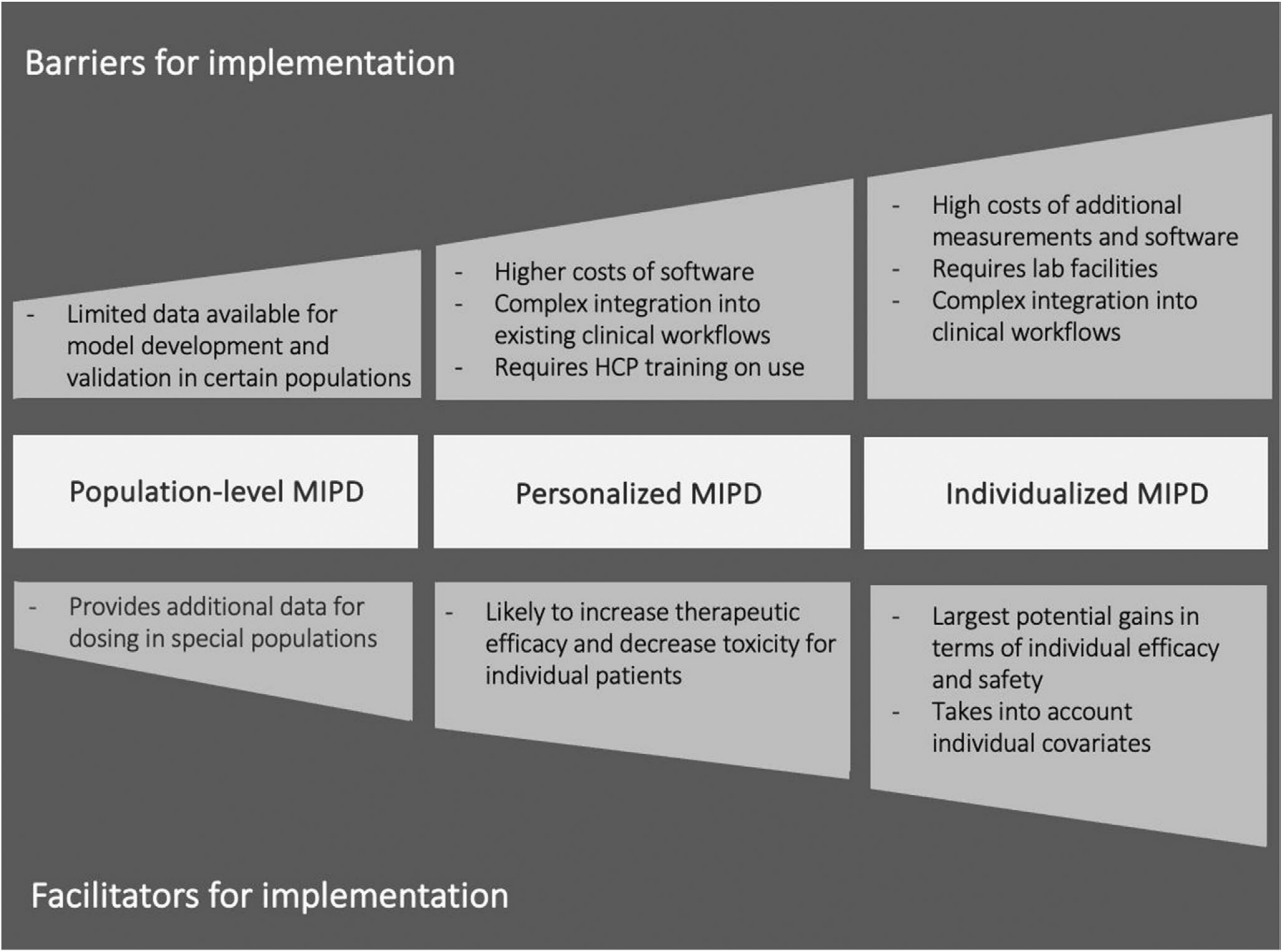
Barriers and facilitators for implementing different MIPD approaches. Abbreviations: HCP: healthcare practitioner. MIPD, model‐informed precision dosing; TDM, therapeutic drug monitoring.

Most articles highlighted a combination of barriers and facilitators across different categories and subcategories. Some articles described facilitators specifically aimed at addressing the identified barriers, while others concentrated on either barriers or facilitators. Barriers and facilitators most frequently fell into the following subcategories: “quality of evidence and model certainty,” “available resources,” “knowledge building,” “regulatory aspects,” and “hosting application.” Collected data are outlined in **Table** **3** (barriers and facilitators by (sub)category) and in **Table** **S2** (barriers and facilitators per article). **Figure** **3** lists differences in the barriers and facilitators identified in this review for the three covered approaches to MIPD.

**Table 3:** Categorized barriers and facilitators

Domain	Category	Subcategory	Barriers	Facilitators
Innovation	Credibility & verifiability	Quality of evidence & model certainty	Clinical datasets contain limited number of a certain subgroup, which may hinder accurate assessment of their characteristics^34^ Suboptimal quality and transparency of models due to limited data for validation^2^, ^20^, ^33^, ^34^, ^36^ Assay errors or incorrectly recorded dosing or sampling could result in inadequate dose recommendations^31^	Evaluation of predictive ability before clinical application of model^34^ Using continuous updates to keep the MIPD tool up to date^18^, ^20^, ^22^, ^31^, ^32^, ^33^, ^34^, ^35^, ^37^, ^39^ Use of independent investigators reporting successes and failures of the software to assure quality^37^ Qualification can be done using historical data from clinical records^34^ Evaluation of model by trained HCP^39^
	Complexity		Complexity of software and models that are often impractical for clinicians to use^35^, ^36^	
Users & stakeholders	Attitude	HCP mindset	Low trust in MIPD approaches^20^, ^36^, ^39^ HCPs still believe in a “one‐size‐fits‐all” approach to dosing^38^	
	Collaboration		Collaboration often restricted between local academia and centers^2^, ^37^	Multistakeholder collaboration to validate, implement and/or demonstrate the value of precision dosing tool^2^, ^20^, ^22^, ^33^, ^37^
	Awareness‐raising & education	Comprehensibility	Cultural differences between HCP and modeling community hampers exchange of knowledge^2^ Different uses of terminology and definitions^20^	
		Knowledge	Little knowledge of PK/PD and the use of models among clinicians^22^, ^31^, ^39^ Lack of relevant training in current medical curriculums^16^, ^20^, ^39^ MIPD is restricted to trained HCPs in specialized centers^2^, ^16^	Increase awareness and transfer knowledge between institutions, researchers, industry and patient groups^2^ Educate and train end‐users^34^, ^36^, ^39^ Increase awareness of advantages of tailored dosing for therapeutic effects^37^ Incorporate education and training on MIPD in medical curriculums and continuous education, for example, offer annual webinar series and hands‐on training^20^, ^32^, ^39^
	Work routine		MIPD not easily integrated in work routine^32^, ^37^, ^38^	Frontline support from clinical pharmacists may be necessary for HCPs who are beginning to utilize MIPD tools^32^ Integrate MIPD tools into EHRs^32^
Implementation	Relevance	Clinical value	MIPD may not always be beneficial compared to standard (TDM‐driven) data^18^	Include data analytics to evaluate the clinical benefit of drug dosing CDS tools^36^ Provide proof of efficacy, reduced toxicity and/or costs^2^, ^27^, ^32^, ^34^, ^36^, ^39^ Demonstrate benefits of MIPD to patients^37^
		Medication selection	Not all medications are suitable for MIPD, for example, low treatment costs or low risk on adverse events^20^, ^32^, ^33^, ^34^	Prioritize medications with high clinical utility^38^
	Feasibility	Regulatory/legal aspects	Lack of clarity on regulatory pathways to endorse use of MIPD^20^, ^36^, ^37^ Liability is uncertain for CDS software^33^	
		Available resources	IT specialists required to integrate MIPD tool into EHRs^32^ Software systems require testing and IT certification^39^ Low availability of medication‐specific formulations and dose strengths to implement the predicted doses^2^ Requires real‐time measurements, but not all facilities are equipped for routine testing and/or analysis^31^ Relative scarcity of point‐of‐care assays and biomarkers^20^ The need for blood sampling and rapid sample measurement availability adds complexity to clinical workflows^16^, ^20^, ^32^	Development of formulations allowing dose individualization^33^ Mail samples overnight to facilities and return the results electronically^31^ Using new CDS tools to automate Bayesian adaptive processes may address staff shortages^31^
		Economic feasibility	Expensive software licenses^15^, ^20^ Training of HCPs is time‐consuming, costly and labor‐intensive^15^	Re‐purposing models used in drug development for clinical application would accelerate MIPD in clinical practice^37^ High costs of overdosing or expensive compounds will be reduced^2^
		Sustainability	Evidence of cost–benefit of MIPD is lacking^2^, ^20^, ^39^	More tools will become available when there is a higher need and use for precision dosing^20^
	Acceptability	Evidence generation	Little published evidence of large‐scale utility^22^, ^39^	Generate a proof of concept for MIPD^2^ Use real‐world evidence to generate clinical evidence for model validation^37^ Publish and share model (recommendations) for widespread evaluation and use^33^
		Patient data	Transferring sensitive patient data across different sources raises challenges^18^, ^20^ Access to individual patient data is complex given data protection laws^18^	Engage patient groups to increase awareness of benefits of MIPD^38^
		Safety safeguards		Warning messages to alert HCPs on potential toxicity^22^, ^31^ Errors corrected or flagged in CDS tool^22^
		Quality improvement		User feedback to inform and update quality improvement processes^31^, ^32^, ^33^
	Access & usability	Hosting application	No user‐friendly tool for integrating data with models to identify optimal dosing^32^	Develop an “easy to integrate in clinical workflow” CDS tool^36^ Easy database searches and data entry for HCPs^16^, ^20^, ^22^, ^38^ Data presented concisely and in chronological order^22^ Availability of an online discussion forum or helpdesk for software users^22^ PopPK incorporated into a clinician‐friendly, easy‐to‐use excel calculator tool^35^
		Integration in healthcare technology		Access from any computer in hospital or remote login^32^, ^39^ Integrate in MIPD tool in EHR^16^, ^32^, ^33^, ^39^ MIPD tool available on mobile devices^33^, ^37^

CDS, clinical decision support; EHR, electronic health record; HCP, healthcare practitioners; IT, information technology; MIPD, model‐informed precision dosing; PD, pharmacodynamics; PK, pharmacokinetics; TDM, therapeutic drug monitoring.

### Innovation

#### Credibility and verifiability

Several studies reported barriers and facilitators pertaining to the credibility and verifiability of models used for MIPD. A frequently mentioned barrier for MIPD implementation was related to challenges surrounding quality assurance due to limited data for model validation.^2^, ^20^, ^33^, ^34^, ^36^ Keizer et al.^34^ also highlighted the need to evaluate the predictive capabilities of a model before its clinical application and suggested that historical data be used to evaluate model performance.

Clinical datasets often contain a limited number of representatives from certain patient subgroups, which may hinder accurate assessment of the impact of certain subgroup characteristics.^34^ Another barrier pertained to the possibility of assay errors being inputted in MIPD tools, potentially resulting in incorrect dose recommendations.^36^ Prior to integrating a model into clinical practice, testing and certification of software systems by information technology experts or trained healthcare practitioners (HCPs) or independent investigators reporting successes and failures of the software, could enhance model credibility.^37^ Continuously updating MIPD tools and their parameters after their implementation in clinical care could significantly improve their effectiveness and quality.^18^, ^20^, ^22^, ^31^, ^32^, ^33^, ^34^, ^35^, ^37^, ^39^

#### Complexity

Additionally, two studies highlighted the complexity of both modeling software and the models themselves, hindering the practical application of MIPD by HCPs.^35^, ^36^ User‐friendly interfaces has been named as an important facilitator to overcome this challenge.^2^, ^20^, ^22^, ^32^, ^33^, ^35^, ^37^ User‐friendly interfaces are particularly important for personalized and individualized MIPD, whereas population‐level MIPD does not require such interfaces for use.

### Users and stakeholders

#### Attitude

Several articles underlined a low trust in MIPD approaches among end‐users including medical doctors and pharmacists, which was attributed to a lack of knowledge as well as limited transparency of MIPD tools.^20^, ^36^, ^37^ Polasek & Shakib^38^ stated that some HCPs viewed precision medicine merely as fine‐tuning for a small number of patients, rather than a game‐changer for all and still believed in a “one‐size‐fits‐all” approach to dosing.

#### Collaboration

Collaboration in the implementation of MIPD was often confined to local academia and healthcare centers.^2^, ^37^ A large number of studies emphasized the need for multistakeholder collaboration across multiple sites, including funding institutions, academia, healthcare professionals, the pharmaceutical industry, regulatory agencies, and patient groups, to validate, implement, and demonstrate the value of MIPD in clinical practice.^2^, ^20^, ^22^, ^27^, ^33^ This collaboration may increase HCPs' awareness of MIPD and of its added value to clinical care.

#### Awareness‐raising and education

Multiple articles noted that disparities in expertise, language, and culture between HCPs and the modeling community hampers exchange of knowledge,^2^ which results in a lack of understanding of the usefulness of models,^22^, ^31^, ^39^ and the utility of MIPD in CDS tools by clinicians.^36^ Currently, training on these topics is not integrated in medical curriculums.^16^, ^22^, ^28^ Additionally, varying terminologies and definitions linked to MIPD may render the topic less comprehensible for HCPs.^20^ This results in MIPD only being used by a limited number of trained HCPs in specialized centers, thus limiting its reach to a small number of patients.^2^, ^16^

Education and training of end‐users^28^, ^34^, ^36^ by incorporating education in medical curriculums or by offering webinar series and hands‐on training may help increase HCPs' knowledge of MIPD.^39^ Enhancing awareness and transfer knowledge between institutions, researchers, the industry, and patient groups could help disseminate the use of MIPD more widely.^2^

#### Work routine

MIPD is not easily integrated into HCPs' work routines, as the interpretation of drug concentrations can be challenging and MIPD tools often require manual data input.^32^, ^37^, ^38^ According to Frymoyer et al., frontline support from clinical pharmacists may be necessary for HCPs who are beginning to use MIPD tools and may require assistance. Nevertheless, given the limitations in personnel and financial resources within most healthcare institutions, achieving this support may be challenging.^32^

### Implementation

#### Relevance

A large number of studies highlight it would be beneficial to showcase evidence demonstrating the efficacy of MIPD in increasing therapeutic effectiveness, reducing toxicity, and/or costs.^2^, ^32^, ^34^, ^36^, ^38^, ^39^

As highlighted by multiple studies, not all medications are suitable for MIPD,^20^, ^32^, ^33^, ^34^ and MIPD may not always be beneficial compared to standard (TDM‐driven) data.^18^ Prioritizing medications with a high utility could make MIPD more clinically relevant, for example drugs that are difficult to dose or medications for patients with complicated needs.^38^ In addition, MIPD could significantly reduce costs by preventing overdosing or unnecessary use of expensive compounds. Data analytics may be used to evaluate the clinical benefit of drug dosing CDS tools.^36^

#### Feasibility

The sampled literature also suggested that regulatory barriers hinder MIPD implementation. Three studies found a lack of clarity on regulatory pathways to endorse the use of MIPD in clinical practice, in both the United States (US) and in European Union (EU).^20^, ^36^, ^37^ Alongside regulatory challenges, legal liability around CDS software and patient‐focused software applications related to MIPD remains uncertain.^33^

Alongside regulatory difficulties, the clinical implementation of MIPD is hindered by the limited availability of various resources to support its use. Software licenses needed for acquiring MIPD tools may be expensive, which makes MIPD less accessible for healthcare facilities with limited resources.^15^, ^20^ Educating staff on the use of MIPD is time‐consuming, costly, and labor‐intensive.^15^ This was mainly noted for personalized and individualized MIPD tools where individual patient data entry was required.^15^, ^20^ Implementing dose recommendations derived from MIPD may also pose challenges due to the limited range of available medication formulations and dose strengths.^2^ To solve this problem, development of drug formulations that allow individualized dosing regimens appears crucial.^33^

Furthermore, MIPD implementation, especially when using TDM, implies additional resources and patient visits dedicated to blood sampling. It may also require new tools or procedures for drug concentration measurements^20^, ^22^ which not all facilities may be equipped to perform. This may be solved by mailing the samples overnight to another facility with the required analytical capabilities.^31^

Given the large resources implications of implementing MIPD in various clinical settings, and the challenges involved, it is crucial to gather evidence regarding the cost‐effectiveness of MIPD in clinical care to enhance its sustainability.^2^, ^20^, ^39^

#### Acceptability

There is little published evidence of the large‐scale utility of MIPD.^22^, ^39^ Darwich et al. propose to build a proof‐of‐concept for MIPD to generate a critical mass of evidence that can encourage wider adoption in clinical care.^2^ Newly developed models or model‐informed dose recommendations must be published and shared for widespread evaluation and use.^33^ To minimize the risk of adverse effects, two studies suggest warning messages to alert HCPs on potential toxicity.^22^, ^31^ Integration of safety safeguards may increase the credibility of model‐informed dose recommendations among HCPs. In addition, demonstrating the benefits of MIPD to patients may help broaden support for MIPD implementation.^37^

#### Access and usability

Several articles highlighted the importance of an easily understandable and user‐friendly interface to facilitate MIPD use,^20^, ^22^, ^32^, ^33^, ^35^, ^37^ as well as uncomplicated database searches and data entry for individualized MIPD tools.^16^, ^20^, ^22^ Data within MIPD tools should be presented concisely and in chronological order, and errors should be corrected or flagged in the CDS tool.^22^ According to Maxfield et al., creating a user‐friendly CDS tool that aligns with HCPs' workflow, may make it easier to incorporate in clinical care. Moreover, ensuring accessibility from any hospital computer or through remote login,^32^, ^39^ as well as availability on mobile devices^33^, ^37^ is recommended. Furthermore, the availability of an online discussion forum or helpdesk for software users to seek assistance if needed has been suggested.^22^

Incorporating MIPD into EHRs may also greatly enhance the adoption of MIPD tools in clinical practice.^16^, ^32^, ^33^, ^39^ This can be done through various approaches: for example, when a prescriber selects a treatment, patient information in the EHR could be integrated with the best practice knowledge embedded in the CDS tool. This integration would enable the prescriber to select the optimal drug and dose regimen and receive clinically important warnings for significant risks.^33^ However, incorporating patient information into MIPD tools could pose challenges due to the complexity of transferring sensitive patient data across multiple sources and data protection laws.^18^, ^20^

## DISCUSSION

This article presents a systematic literature review regarding the barriers and facilitators for the clinical implementation of MIPD.

### Main findings and implications for practice

Several barriers may account for the limited implementation of MIPD in clinical practice. These include restricted data for model validation, raising challenges for quality assurance, as well as limited transparency regarding model assumptions towards users. Data availability may vary depending on the patient population and the date of drug licensing. In the absence of a legal mandate to clinically investigate a drug in certain patient populations for licensing, available data for model validation may be particularly limited in these populations,^20^ including pregnant women.

Another major barrier pertains to the financial burden of implementing MIPD for hospitals, which often operate with limited resources. This may include the costs of additional training for HCPs, conducting point‐of‐care measurements, and the procurement of expensive software licenses. Furthermore, the varying levels of trust in MIPD among HCPs may partially result from their limited knowledge of pharmacology and existing modeling approaches. In this context, easily understandable and user‐friendly interfaces may be seen as a critical way of facilitating a successful adoption of MIPD in clinical care. Continuous updates of the models being deployed, based on newly generated data as part of their clinical use, could increase both the credibility and clinical utility of these tools. Additionally, education and training of end‐users on MIPD approaches may lead to increased trust in these approaches. Lastly, multistakeholder collaboration could greatly enhance the implementation of MIPD in clinical care.

Many of the barriers and facilitators identified in this review aligned with factors highlighted in studies focusing on the implementation of other types of precision medicine in clinical care. Examples of such approaches include artificial intelligence (AI) and pharmacogenomics (PGx). Studies about the clinical implementation of AI and PGx may also offer new insights for the dissemination of MIPD.

In line with our findings regarding MIPD credibility and user trust, AI models have been called “black boxes” due to their inability to explain their recommendations. The development of transparent AI models, where users can access information regarding the reasoning behind outlined recommendations, could help identify biases in these models^40^ and enhance their credibility among HCPs.^41^ Furthermore, rigorous validation of AI systems is essential to ensure accuracy. Aligning with our results, a scoping review on the clinical implementation of AI underlined the importance of involving both information and communication technology and clinical domain experts for implementing AI into clinical practice. This review also suggested that AI models adding to HCPs workload would be much less likely to be used. Integration of AI into CDS tools already being used was found to ease integration into HCPs' workflows.^42^

Looking at PGx, a recent report by the British Royal College of Physicians and the British Pharmacological Society detailed several barriers and steps for disseminating the use of this approach in a clinical context.^43^ In line with our findings and those on AI, it highlighted prescribers' limited knowledge of pharmacogenomics as a key obstacle to address. PGx trainings should be integrated into medical and pharmaceutical curriculums, and learning resources be made available at or near the point of prescribing. In addition, this report highlighted the need for pharmacogenomic research to be conducted collaboratively, inclusively, and across disciplines.^44^ Alongside varying levels of understanding of PGx and its significance among HCPs, patients, and the public, a literature review by Klein et al. highlighted the lack of configuration of EHRs to deal with genetic information of patients as an additional barrier for PGx implementation. Strategies to address these hurdles included improving EHRs to receive, store, and present complex genomic information for clinical use, incorporating PGx lectures into HCPs' training and the development of guidelines describing the utility of PGx testing to clinicians.^45^

Examining regulatory barriers, existing regulations may pose greater challenges for the clinical application of personalized and individualized MIPD tools compared to population‐level MIPD. In the EU, the Medical Devices Regulation (MDR) requires that medical devices such as medical software undergo a conformity assessment to demonstrate that they meet legal requirements around safety.^45^ This may entail significant time and expenses from device manufacturers.^46^ The MDR primarily constitutes a barrier to personalized and individualized MIPD tools, which generally qualify as medical devices due to the transformation of individual patient data implied.^45^ In principle, population‐level MIPD remains exempt from MDR compliance.

While many of the barriers and facilitators identified in this review were broadly applicable to the clinical implementation of MIPD, some were more specifically relevant to certain types of precision dosing. This was particularly true for individualized MIPD, implying greater logistical as well as regulatory challenges than standardized, population‐level model‐informed doses. “A priori” dose adjustments may require manual input of individual patient characteristics, which may be time‐consuming. Individualized MIPD with a posteriori data introduces even greater complexity and logistical hurdles by requiring real‐time measurements, additional blood sampling, rapid sample measurement availability and staff to interpret the data. Furthermore, integrating the software required for determining personalized doses into EHRs presents both technical and regulatory challenges, particularly due to the diversity of EHR systems used across hospitals and, in regions such as the EU and US, the need to comply with medical device regulations.

Variations in identified barriers and facilitators for various MIPD approaches underscore the importance of carefully choosing the most fitting MIPD method for addressing a given clinical need. They also entail different strategies for implementation in a clinical context.

Different stakeholders could be engaged to support efforts aimed at the implementation of MIPD in clinical settings. Companies developing MIPD tools should prioritize the user‐friendliness of these tools to enhance their usability in clinical practice. Clinical pharmacology societies could develop educational materials aimed at clinicians to expand their knowledge on pharmacokinetic modeling. Additionally, universities might consider incorporating education on MIPD into medical curriculums to ensure that HCPs possess at least a basic knowledge of pharmacokinetic models. The newly created and openly accessible MELINDA website (ModEL‐Informed Dosing for All, https://www.melinda‐dosing.com) aims to educate clinicians about MIPD and its potential added value in clinical care. Finally, organizing workshops on pharmacokinetic modeling for clinicians and patients with limited familiarity with modeling could improve their understanding of these models.

### Strengths and limitations

To our knowledge, this is the first systematic review of the literature on factors that may influence the clinical implementation of MIPD. Other strengths include the broad definition of MIPD, and the various approaches covered, with no limitation on the type of model, patient population, or therapeutic area. We drew on a comprehensive analytical framework to capture a broad range of relevant barriers and facilitators and stratified these factors according to the type of MIPD approach used.

The quality of included studies appeared sufficient. In most cases, the first authors were pharmacometricians or pharmacists. This may imply a risk of bias, with MIPD experts possibly holding a more positive view of MIPD than average HCPs, thus potentially missing barriers for implementation. Nevertheless, most of the identified barriers and facilitators stemmed from qualitative studies exploring the perspectives of HCPs regarding the pilot implementation of a specific MIPD tool in clinical practice. This may enhance the reliability of these studies in capturing HCPs' perceptions of MIPD. The diversity of models examined increased the probability of an unbiased reflection of the field's perspectives on the clinical use of MIPD.

Several limitations apply. Searching only one literature database may have led to missing relevant studies. Second, although the type of modeling used for MIPD did not affect study eligibility, our search string primarily focused on popPK and PBPK models. Broader search terms such as “precision dosing” were also employed. Studies examining other types of models used for MIPD, such as PK/PD models or AI‐learning algorithms, may have been missed. Most included studies examined individualized and personalized MIPD, indicating that relevant factors were at least partially captured. Third, studies that did not primarily focus on the clinical implementation of MIPD, but rather concentrated on aspects such as model development and validation, were excluded. Excluded articles may have alluded to relevant barriers or facilitators. However, significant overlap in barriers and facilitators was noted across the sampled studies, suggesting that the provided overview was comprehensive. Furthermore, the absence of search terms specifically related to the economic feasibility of MIPD in the search strategy may have contributed to the limited information included in this review. However, several sampled studies discussed constraints pertaining to economic feasibility, and the resources needed for the implementation of MIPD in clinical care. Lastly, the review's focus on peer‐reviewed studies may have resulted in omitting other relevant information about the clinical implementation of MIPD, particularly regarding privately developed and commercialized MIPD tools, thereby potentially limiting the overall understanding of the breadth of MIPD uptake.

The reviewed literature primarily included perspectives, narrative reviews, and expert opinions. This may somewhat limit the generalizability of the findings given the subjective nature of these study designs. Furthermore, different JBI critical appraisal checklists had to be employed for assessing these various studies, complicating the overall quality assessment of sampled reports. Although many of the identified barriers and facilitators likely applied across various MIPD approaches, insights specifically relevant to population‐level MIPD were limited. Furthermore, most included studies examined the use of MIPD to a general patient population. Factors influencing MIPD application to specific patient groups, whether based on their physiology or distinct therapeutic needs, may differ. For example, children with human immunodeficiency virus may be more vulnerable to drug‐related adverse events because of large interindividual variations in plasma concentrations.^47^ Identified barriers and facilitators may also vary across clinical settings, an aspect that received limited scrutiny in this review. Importantly, the sampled articles examined the viewpoints of HCPs, without exploring patients' perspectives. Examining patients' opinions could significantly enrich our understanding of factors influencing MIPD implementation in clinical practice, particularly in the context of shared decision making about drug dosing.^48^ Despite the potential benefits of involving patients in decision making on tailored doses, our review did not identify any instances in which shared decision making regarding drug dosing were reported.

## CONCLUSION

This systematic review identified barriers and facilitators for the clinical implementation of MIPD. Potential hurdles to overcome include unclear regulatory pathways for MIPD validation and application and heterogeneous quality assurance due to limited data for model validation. Collaboration between multiple stakeholders to accelerate the design and validation of MIPD tools could increase the amount of data and models available. Additionally, this review offers useful insights for improving the user‐friendliness and clinical usability of MIPD tools. Addressing the identified barriers through collaborative efforts involving multiple stakeholders and raising awareness about available MIPD tools and their benefits among both HCPs and patients can help accelerate MIPD adoption. This appears critical to maximize the value of MIPD, which can enhance patient outcomes by ensuring their access to tailored medication therapy.

## FUNDING

This publication is based on research funded by the Bill & Melinda Gates Foundation (INV‐023795). The findings and conclusions contained within are those of the authors and do not necessarily reflect positions or policies of the Bill & Melinda Gates Foundation.

## CONFLICT OF INTEREST

Dr de Wildt receives compensation for consultancy work for Khondrion. All other authors declared no competing interests for this work.

## AUTHOR CONTRIBUTIONS

C.D., C.K., and E.O. wrote the manuscript. C.D., C.K., E.O., H.S., and S.N.W. designed the research; C.D. and E.O. performed the research; C.D., C.K., and E.O. analyzed the data.

## Supporting information


Table S1.
