# Supplementary material for: Barriers and Facilitators for Bringing Model‐Informed Precision Dosing to the Patient's Bedside: A Systematic Review
Source: Clin Pharmacol Ther. 2024 Dec 10;117(3):633–45. doi: 10.1002/cpt.3510 (PMC11835426; doi:10.1002/cpt.3510)
Supplement: Supplementary file 1 — Table S1. [file CPT-117-633-s001.docx]

**Supplementary materials:**

S1. Search string used for the search in PubMed p. 2

S2. Barriers and facilitators identified in each article p. 2-10

**Table S1: Search string used for the search in PubMed**

| **Search** |
| --- |
| (("model informed dos*"[Title/Abstract]) OR ("personalised dos*"[Title/Abstract]) OR ("personalized dos*"[Title/Abstract]) OR ("precision dos*"[Title/Abstract]) OR ("model based dos*"[Title/Abstract]) OR ("dosing algorithm"[Title/Abstract]) OR ("individualized dos*"[Title/Abstract]) OR ("individualised dos*"[Title/Abstract]) OR ("rational dos*"[Title/Abstract]) OR ("physiologically based pharmacokinetic model*"[Title/Abstract]) OR ("physiology based pharmacokinetic model*"[Title/Abstract]) OR ("PBPK model*"[Title/Abstract]) OR (''population pharmacokinetic model*''[Title/abstract]) AND  ((Patient*[Title/Abstract) OR (Clinic*[Title/Abstract) OR (Healthcare[Title/Abstract) OR (Care[Title/Abstract) OR (Therap*[Title/Abstract)) AND ((implement*[Title/Abstract]) OR (framework[Title/Abstract]) OR (barrier*[Title/Abstract]) OR (regulator*[title/abstract]) OR (opportunit*[Title/Abstract]) OR (challenge*[Title/Abstract])) NOT ("drug development"[Title/Abstract]) NOT ("drug drug interaction"[Title/Abstract]) NOT (Animals[MeSH] NOT Humans[MeSH]) |

**Table S2: Barriers and facilitators identified in each article**

| Study | Identified category | Barriers | Facilitators |
| --- | --- | --- | --- |
| Darwich et al. 2017^2^ | ***Innovation***  *Credibility & verifiability*  ***Users & stakeholders***  *Collaboration*  *Awareness-raising & education*  ***Implementation***  *Relevance*  *Feasibility*  *Acceptability* | - Quality of evidence & model certainty: suboptimal quality and transparency of models due to limited data for validation.  - Collaboration often restricted between local academia and centers  - Comprehensibility: cultural differences between HCP and modelling community hampers exchange of knowledge  - Knowledge: MIPD is restricted to trained HCPs in specialized centers  - Available resources: low availability of medication-specific formulations and dose strengths to implement the predicted doses  - Sustainability: evidence of cost-benefit of MIPD is lacking | - Multistakeholder collaboration to validate, implement and/or demonstrate the value of precision dosing tool  - Knowledge: increase awareness and transfer knowledge between institutions, researchers, industry and patient groups  - Clinical value: provide proof of efficacy, reduced toxicity and/or costs  - Economic feasibility: High costs of overdosing or expensive compounds will be reduced  - Evidence generation: generate a proof of concept for MIPD |
| Euteneuer et al. 2019^31^ | ***Innovation***  *Credibility & verifiability*  ***Users & stakeholders***  *Awareness-raising & education*  ***Implementation***  *Feasibility*  *Acceptability* | - Quality of evidence & model certainty: assay errors or incorrectly recorded dosing or sampling could result in inadequate dose recommendations  - Knowledge: little knowledge of PK/PD and the use of models among clinicians  - Available resources: requires real-time measurements, but not all facilities are equipped for routine testing and/or analysis | - Quality of evidence & model certainty: using continuous updates to keep the MIPD tool up to date  - Available resources:  *Mail samples overnight to facilities and return the results electronically  *Using new CDS tools to automate Bayesian adaptive processes may address staff shortages  - Patient data: warning messages to alert HCPs on potential toxicity  - Quality improvement: user feedback to inform and update quality improvement processes |
| Frymoyer et al. 2020^32^ | ***Innovation***  *Credibility & verifiability*  ***Users & stakeholders***  *Awareness-raising & education*  ***Implementation***  *Relevance*  *Feasibility*  *Acceptability*  *Access & usability* | - Work routine: MIPD not easily integrated in work routine  - Medication selection: Not all medications are suitable for MIPD, e.g. low treatment costs or low risk on adverse events  - Available resources: IT specialists required to integrate MIPD tool into EHRs  - Hosting application: no user-friendly tool for integrating data with models to identify optimal dosing | - Quality of evidence & model certainty: using continuous updates to keep the MIPD tool up to date  - Knowledge: incorporate education and training on MIPD in medical curriculums and continuous education, e.g. offer annual webinar series and hands-on training  - Work routine:  *Frontline support from clinical pharmacists may be necessary for HCPs who are beginning to utilize MIPD tools^27^  *Integrate MIPD tools into EHRs  - Clinical value: provide proof of efficacy, reduced toxicity and/or costs  - Quality improvement: user feedback to inform and update quality improvement processes  - Integration in healthcare technology:  *Access from any computer in hospital or remote login  *Integrate in MIPD tool in EHR |
| Gonzalez et al. 2017^33^ | ***Innovation***  *Credibility & verifiability*  ***Users & stakeholders***  *Collaboration*  ***Implementation***  *Relevance*  *Feasibility*  *Acceptability*  *Access & usability* | - Quality of evidence & model certainty: suboptimal quality and transparency of models due to limited data for validation.  - Medication selection: Not all medications are suitable for MIPD, e.g. low treatment costs or low risk on adverse events  - Regulatory/legal aspects: liability is uncertain for CDS software | - Quality of evidence & model certainty: using continuous updates to keep the MIPD tool up to date  - Multistakeholder collaboration to validate, implement and/or demonstrate the value of precision dosing tool  - Available resources: development of formulations allowing dose individualization  - Evidence generation: publish and share model (recommendations) for widespread evaluation and use  - Quality improvement: user feedback to inform and update quality improvement processes  - Integration in healthcare technology:  *Integrate in MIPD tool in HER  *MIPD tool available on mobile devices |
| Kantasiripitak et al. 2020^22^ | ***Innovation***  *Credibility & verifiability*  ***Users & stakeholders***  *Collaboration*  *Awareness-raising & education*  ***Implementation***  *Feasibility*  *Acceptability*  *Access & usability* | - Knowledge: little knowledge of PK/PD and the use of models among clinicians  - Available resources: the need for blood sampling and rapid sample measurement availability adds complexity to clinical workflows  - Evidence generation: little published evidence of large-scale utility | - Quality of evidence & model certainty: using continuous updates to keep the MIPD tool up to date  - Multistakeholder collaboration to validate, implement and/or demonstrate the value of precision dosing tool  - Patient data:  *Warning messages to alert HCPs on potential toxicity  *Errors corrected or flagged in CDS tool  - Hosting application  *Easy database searches and data entry for HCPs  *Data presented concisely and in chronological order  *Availability of an online discussion forum or helpdesk for software users |
| Keizer et al. 2018^34^ | ***Innovation***  *Credibility & verifiability*  ***Users & stakeholders***  *Awareness-raising & education*  ***Implementation***  *Relevance* | - Quality of evidence & model certainty  *Suboptimal quality and transparency of models due to limited data for validation.  *Clinical datasets contain limited number of a certain subgroup, which may hinder accurate assessment of their characteristics  - Medication selection: Not all medications are suitable for MIPD, e.g. low treatment costs or low risk on adverse events | - Quality of evidence & model certainty:  *Evaluation of predictive ability before clinical application of model  *Using continuous updates to keep the MIPD tool up to date  *Qualification can be done using historical data from clinical records  - Knowledge: Educate and train end-users  - Clinical value: provide proof of efficacy, reduced toxicity and/or costs |
| Kluwe et al. 2020^20^ | ***Innovation***  *Credibility & verifiability*  ***Users & stakeholders***  *Attitude*  *Collaboration*  *Awareness-raising & education*  ***Implementation***  *Relevance*  *Feasibility*  Acceptability  Access & usability | - Quality of evidence & model certainty: suboptimal quality and transparency of models due to limited data for validation.  - HCP mindset: Low trust in MIPD approaches  - Comprehensibility: different uses of terminology and definitions  - Knowledge: lack of relevant training in current medical curriculums  - Medication selection: Not all medications are suitable for MIPD, e.g. low treatment costs or low risk on adverse events  - Regulatory/legal aspects: lack of clarity on regulatory pathways to endorse use of MIPD  - Available resources:  *Relative scarcity of point-of-care assays and biomarkers  *The need for blood sampling and rapid sample measurement availability adds complexity to clinical workflows  - Economic feasibility: expensive software licenses  - Sustainability: evidence of cost-benefit of MIPD is lacking  - Patient data: transferring sensitive patient data across different sources raises challenges | - Quality of evidence & model certainty: using continuous updates to keep the MIPD tool up to date  - Multistakeholder collaboration to validate, implement and/or demonstrate the value of precision dosing tool  - Knowledge: incorporate education and training on MIPD in medical curriculums and continuous education, e.g. offer annual webinar series and hands-on training  - Sustainability: more tools will become available when there is a higher need and use for precision dosing  - Hosting application: easy database searches and data entry for HCPs |
| Long-Boyle et al. 2015^35^ | ***Innovation***  *Complexity*  *Access & usability* | - Complexity of software and models that are often impractical for clinicians to use | - Quality of evidence & model certainty: using continuous updates to keep the MIPD tool up to date  - Hosting application: PopPK incorporated into a clinician-friendly, easy-to-use excel calculator tool |
| Maier et al. 2022^18^ | ***Innovation***  *Credibility & verifiability*  ***Implementation***  *Relevance*  *Feasibility*  *Acceptability* | - Clinical value: MIPD may not always be beneficial compared to standard (TDM-driven) data  - Available resources: the need for blood sampling and rapid sample measurement availability adds complexity to clinical workflows  - Patient data:  *Transferring sensitive patient data across different sources raises challenges  *Access to individual patient data is complex given data protection laws | - Quality of evidence & model certainty: using continuous updates to keep the MIPD tool up to date |
| Maxfield et al. 2020^36^ | ***Innovation***  *Credibility & verifiability*  ***Users & stakeholders***  *Attitude*  *Knowledge*  ***Implementation***  *Relevance*  *Feasibility*  *Access & usability* | - Quality of evidence & model certainty: suboptimal quality and transparency of models due to limited data for validation.  - Complexity of software and models that are often impractical for clinicians to use  - HCP mindset: Low trust in MIPD approaches  - Regulatory/legal aspects: lack of clarity on regulatory pathways to endorse use of MIPD | - Knowledge: Educate and train end-users  - Clinical value:  *Include data analytics to evaluate the clinical benefit of drug dosing CDS tools  *Provide proof of efficacy, reduced toxicity and/or costs  - Hosting application: develop an ‘easy to integrate in clinical workflow’ CDS tool |
| Mizuno et al. 2022^16^ | ***Users & stakeholders***  *Awareness-raising & education*  ***Implementation***  *Feasibility*  *Access & usability* | - Knowledge:  *Lack of relevant training in current medical curriculums  *MIPD is restricted to trained HCPs in specialized centers  - Available resources: the need for blood sampling and rapid sample measurement availability adds complexity to clinical workflows | - Hosting application: easy database searches and data entry for HCPs  - Integration in healthcare technology: integrate in MIPD tool in EHR |
| Perry et al. 2020^15^ | ***Implementation***  *Feasibility* | - Economic feasibility:  - Expensive software licenses  - Training of HCPs is time-consuming, costly and labor-intensive |  |
| Polasek et al. 2019^37^ | ***Innovation***  *Credibility & verifiability*  ***Users & stakeholders***  *Collaboration*  *Awareness-raising & education*  ***Implementation***  *Relevance*  *Feasibility*  *Acceptability*  *Access & usability* | - Collaboration often restricted between local academia and centers  - Work routine: MIPD not easily integrated in work routine  - Regulatory/legal aspects: lack of clarity on regulatory pathways to endorse use of MIPD | - Quality of evidence & model certainty:  *Using continuous updates to keep the MIPD tool up to date  *Use of independent investigators reporting successes and failures of the software to assure quality  - Multistakeholder collaboration to validate, implement and/or demonstrate the value of precision dosing tool  - Knowledge: increase awareness of advantages of tailored dosing for therapeutic effects  - Clinical value: demonstrate benefits of MIPD to patients  - Economic feasibility: re-purposing models used in drug development for clinical application would accelerate MIPD in clinical practice  - Evidence generation: use real-world evidence to generate clinical evidence for model validation  - Integration in healthcare technology: MIPD tool available on mobile devices |
| Polasek, Shakib et al. 2019^38^ | ***Users & stakeholders***  *Attitude*  *Awareness-raising & education*  ***Implementation***  *Relevance*  *Acceptability*  *Access & usability* | - HCP mindset: HCPs still believe in a ‘one-size-fits-all’ approach to dosing  - Work routine: MIPD not easily integrated in work routine | - Clinical value: provide proof of efficacy, reduced toxicity and/or costs  - Medication selection: prioritize medications with high clinical utility  - Patient data: engage patient groups to increase awareness of benefits of MIPD  - Hosting application: easy database searches and data entry for HCPs |
| Vinks et al. 2020^39^ | ***Innovation***  *Credibility & verifiability*  ***Users & stakeholders***  *Attitude*  *Awareness-raising & education*  ***Implementation***  *Relevance*  *Feasibility*  *Acceptability*  *Access & usability* | - HCP mindset: Low trust in MIPD approaches  - Knowledge:  *Little knowledge of PK/PD and the use of models among clinicians  *Lack of relevant training in current medical curriculums  - Available resources: software systems require testing and IT certification  - Sustainability: evidence of cost-benefit of MIPD is lacking  - Evidence generation: little published evidence of large-scale utility | - Quality of evidence & model certainty:  *Using continuous updates to keep the MIPD tool up to date  *Evaluation of model by trained HCP  - Knowledge:  *Educate and train end-users  *Incorporate education and training on MIPD in medical curriculums and continuous education, e.g. offer annual webinar series and hands-on training  - Clinical value: provide proof of efficacy, reduced toxicity and/or costs  - Integration in healthcare technology:  *Access from any computer in hospital or remote login  * Integrate in MIPD tool in HER |

*CDS: clinical decision support, EHR: electronic health records, HCP: healthcare practitioners, IT: information technology, MIPD: model-informed precision dosing, PD: pharmacodynamics, PK: pharmacokinetics, TDM: therapeutic drug monitoring*
